# Supplementary material for: p62/SQSTM1-droplet serves as a platform for autophagosome formation and anti-oxidative stress response
Source: Nat Commun. 2021 Jan 4;12:16. doi: 10.1038/s41467-020-20185-1 (PMC7782522; doi:10.1038/s41467-020-20185-1)
Supplement: Supplementary file 1 — Supplementary Information [file 41467_2020_20185_MOESM1_ESM.pdf]

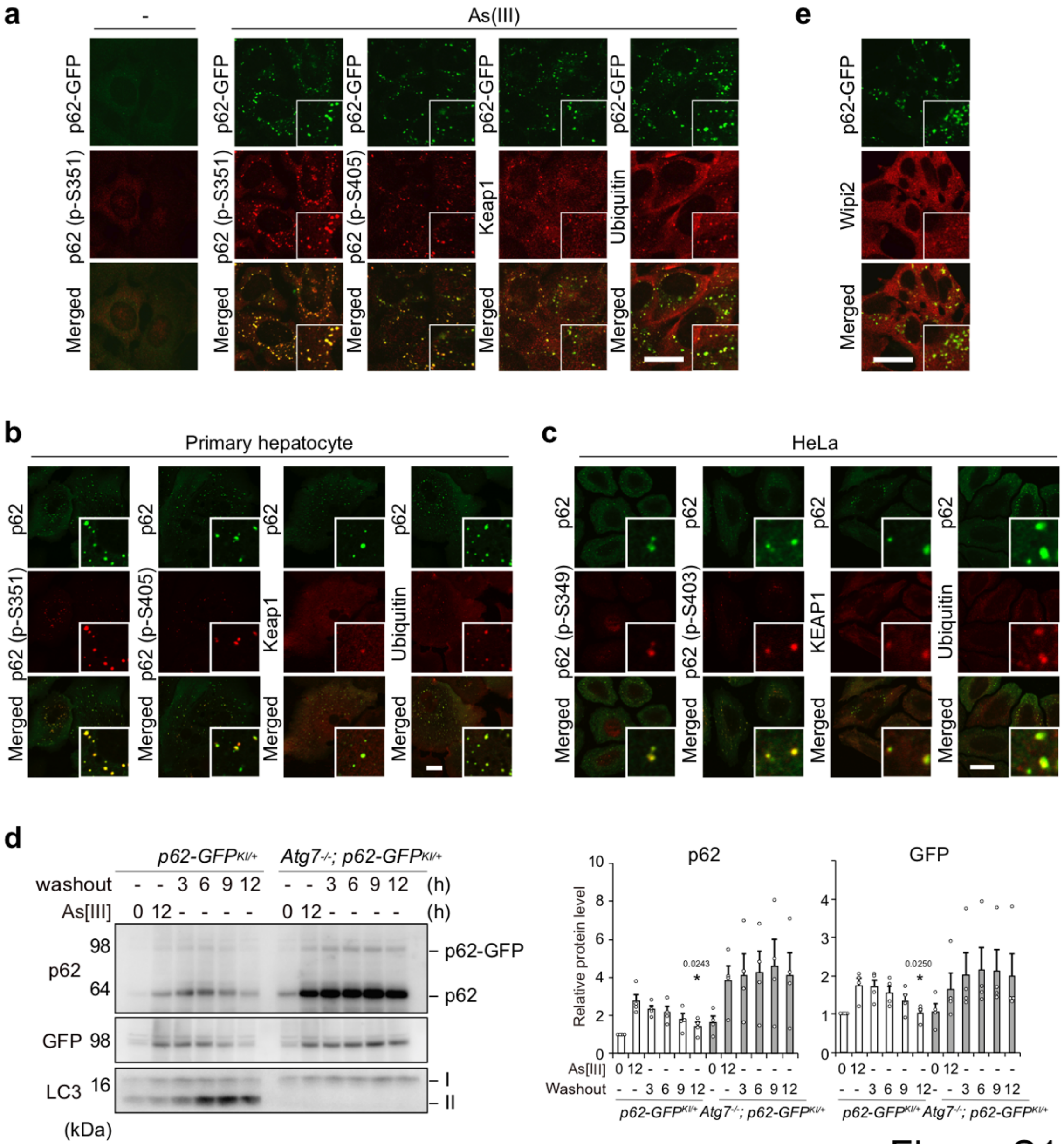

Figure S1

Supplementary Figure S1 Autophagic turnover of p62-gels.
(**a-c**) Immunofluorescence microscopy. *p62-GFP*$^{K/+}$ MEFs (a), primary mouse hepatocytes (b) and HeLa cells (c) were cultured in regular medium or medium containing 10 µM (a and b) and 20 µM (c) arsenite (As[III]) for 10 hr (a) and for 12 hr (b and c). After removal of As[III], cells were cultured in regular medium for 3 hr. The cells were immunostained with indicated antibodies. Bars: 20 µm.
(d) Immunoblot analysis. *p62-GFP*$^{KI/+}$ and *Atg7*$^{-/-}$;*p62-GFP*$^{KI/+}$ MEFs were challenged by As[III]. After removal of As[III], cells were cultured in regular medium for the indicated time. Cell lysates were prepared and subjected to immunoblot analysis with the specified antibodies. Data shown are representative of four separate experiments. Bar graphs indicate the quantitative densitometric analysis of the indicated proteins relative to whole proteins estimated by Ponceau-S staining (n =4). Data are means ± s.e. *P < 0.05 as determined by two-sided Welch's t-test.

(**e**) Immunofluorescence microscopy. *p62-GFP*$^{K/+}$ MEFs were cultured as described in (a) and then immunostained with WIPI2 antibody. Bars: 20 µm. Source data are provided as a Source Data file.

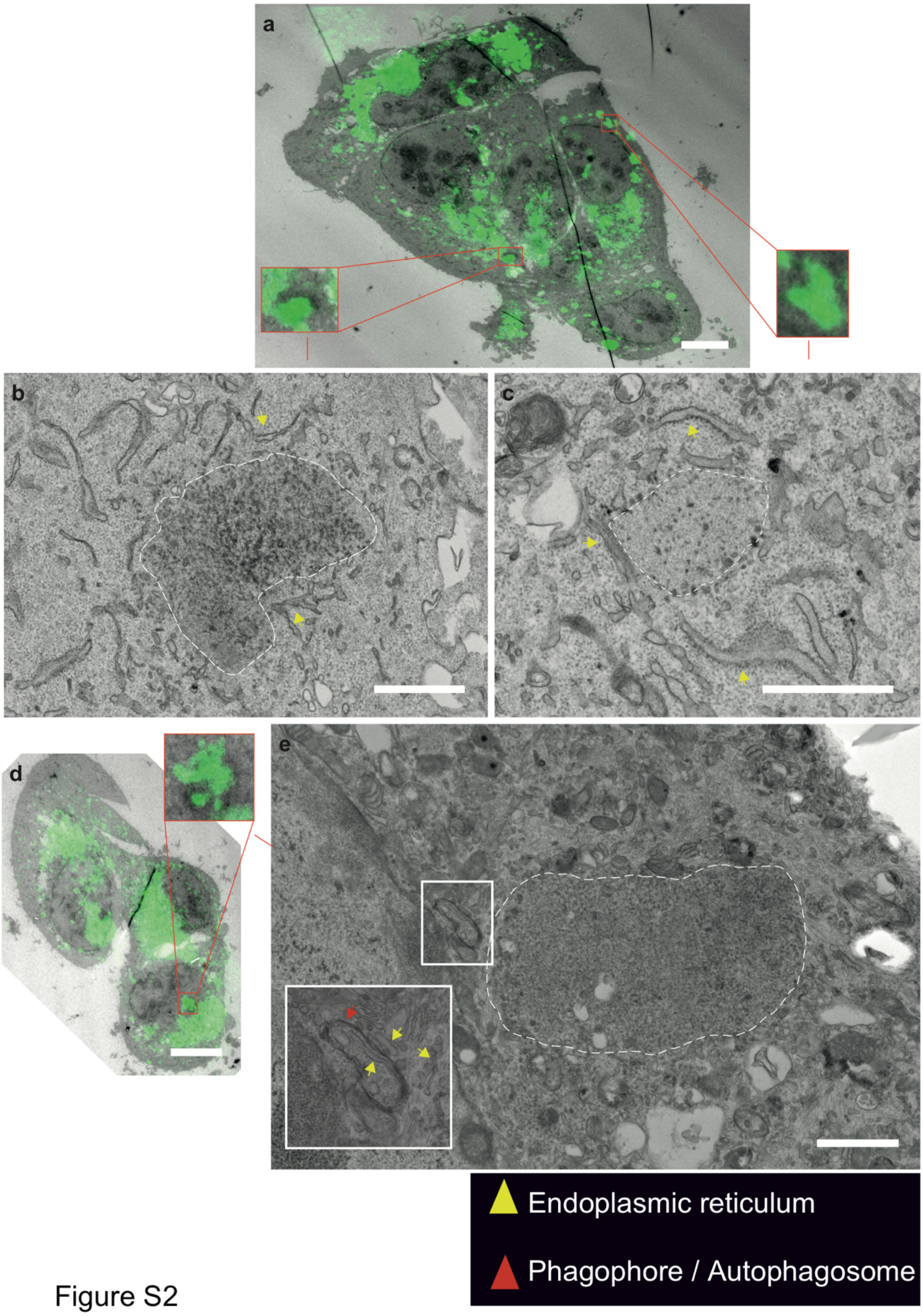

Figure S2

Supplementary Figure S2 Electron microscopy of p62 gels in *Atg7*-deficient MEFs. (**a**, **d**) Correlation of GFP fluorescence and TEM. (**b**, **c**) p62-gels, indicated by the dotted line, are surrounded by endoplasmic reticulum (yellow arrows). (**e**) p62-gel, indicated by the dotted line, and an adjacent phagophore/isolation membrane or autophagosome (red arrowhead). The

insert shows a higher magnification of the boxed area in panel **e**. Note that the phagophore/isolation membrane or autophagosome does not engulf p62. The cells were treated as in Figure 2. Bars: 10 μm (a and d) and 1 μm (b, c and e).

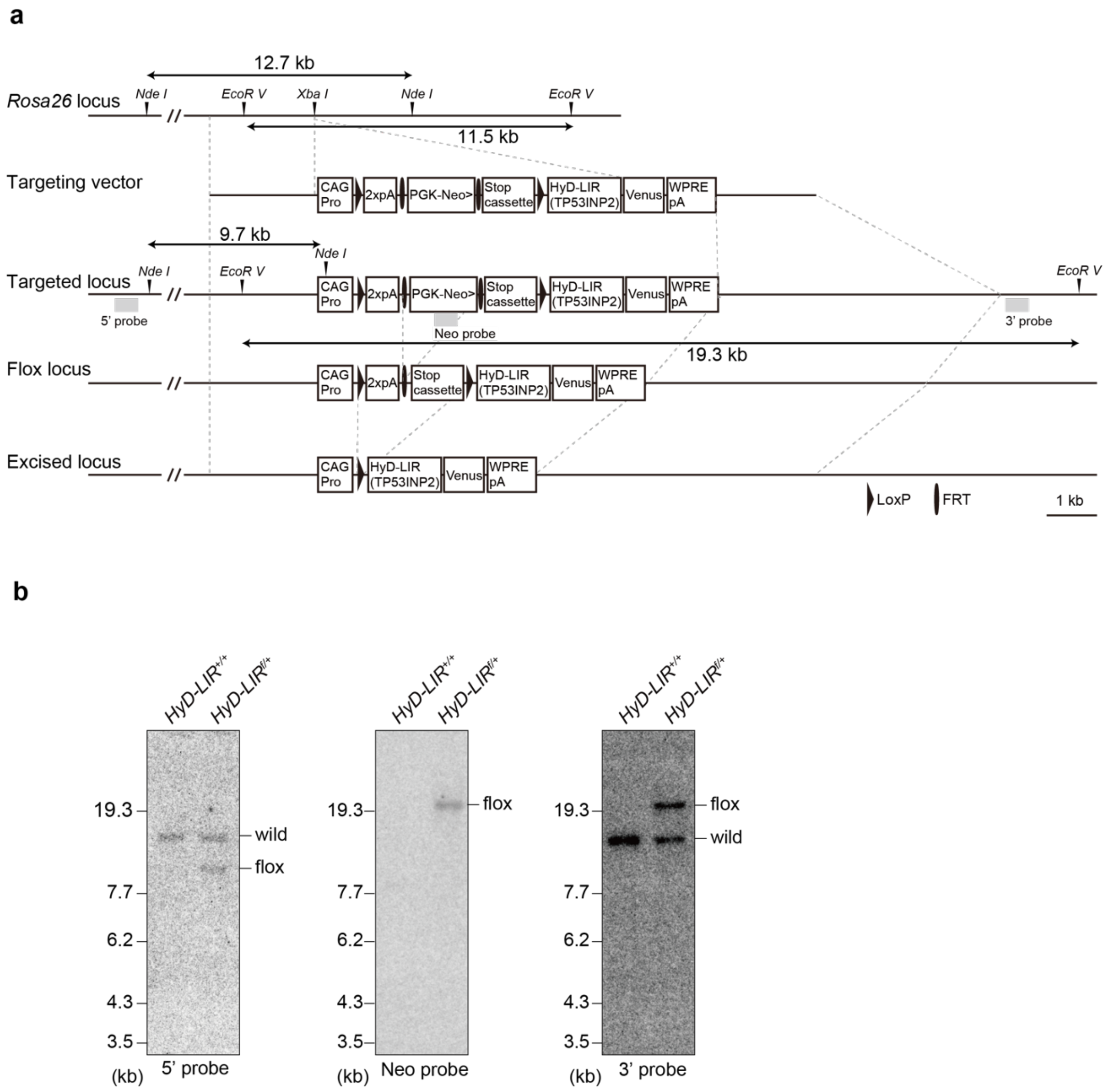

Figure S3

Supplementary Figure S3 Generation of *HyD-LIR-Venus* knock-in mice

(**a**) Schematic representation of knock-in mice that express HyD-LIR-Venus under CAG promoter in a Cre-recombinase dependent manner.

(**b**) Southern blot analysis. Southern blot analysis of genomic DNA extracted from mouse tail. Wild-type and flox alleles are detected as 12.7- and 9.7-kb bands with 5' probe after NdeI digestion or 11.5- and 19.3-kb bands with 3' probe after EcoRV digestion, respectively. Flox allele is detected as 19.3-kb bands with 3' probe after NdeI digestion.

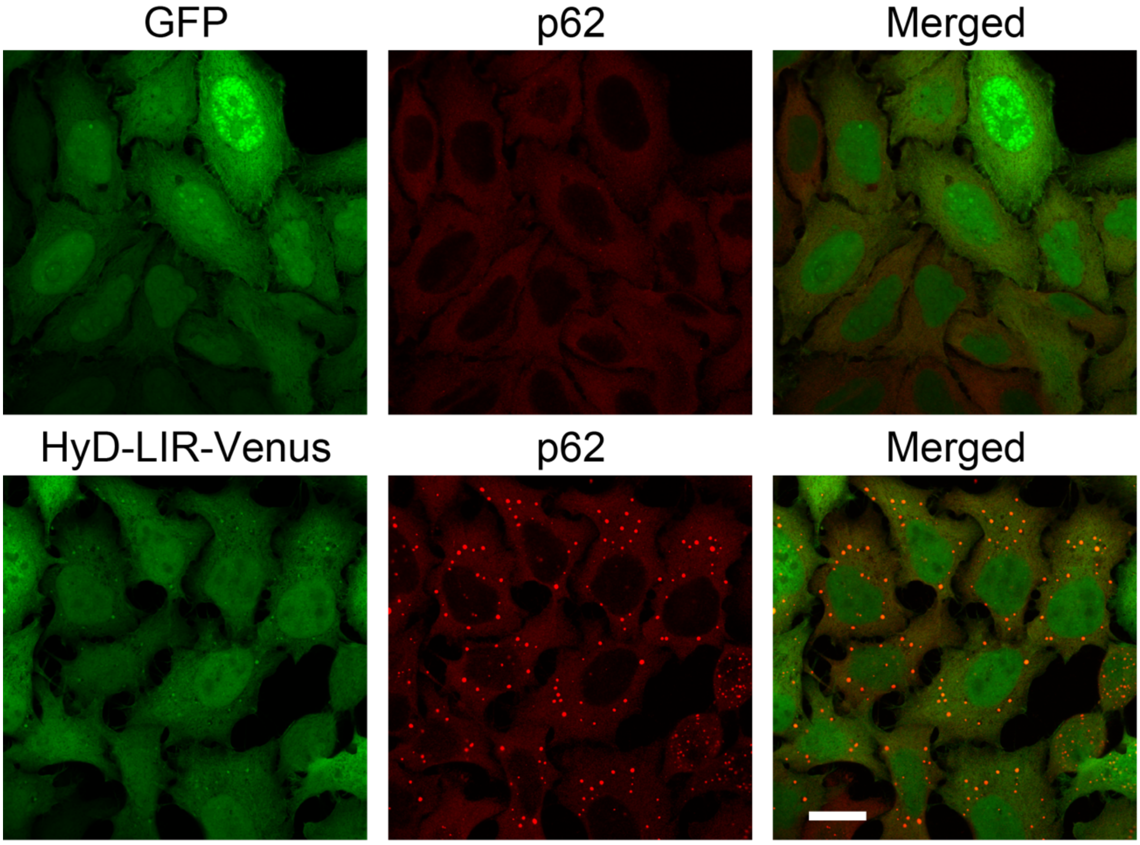

Figure S4

Supplementary Figure S4 The accumulation of p62-gels in HeLa cells expressing HyD-LIR-Venus

HeLa cells were infected with adenovirus GFP or HyD-LIR-Venus. 48 hr after the infection, the cells were immunostained with p62 antibody. Bar: 20 µm.

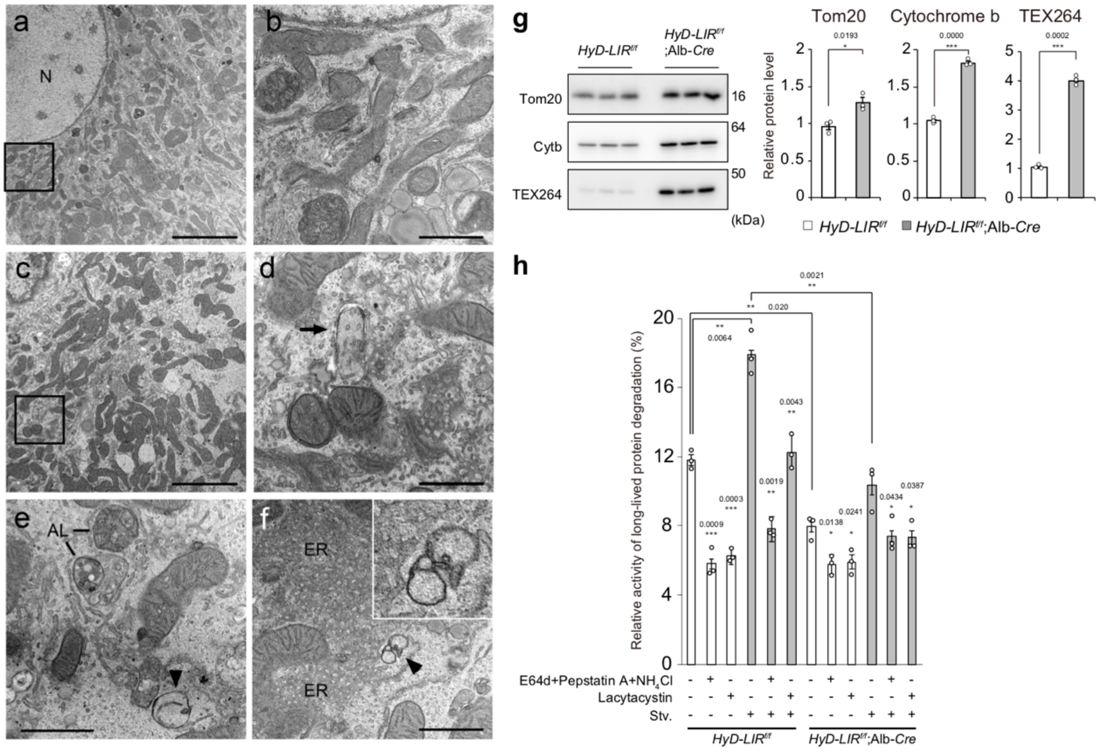

Figure S5

Supplementary Figure S5 The accumulation of mitochondria and endoplasmic reticulum in hepatocytes expressing HyD-LIR-Venus

(**a-f**) Electron microscopy. Representative electron micrographs of hepatocytes from *HyD-LIR*$^{flox/flox}$ and *HyD-LIR*$^{flox/flox}$; Alb-*Cre* mice. Boxed regions in (a) and (c) are magnified and shown in (b) and (d), respectively. The region of arrowhead in (f) is magnified and shown in the inset. Arrow: phagophore, Arrowheads: autophagosome/ phagophore profiles. ER: endoplasmic reticulum, AL: autolysosome, Bars: 5 µm (a and c) and 1 µm (b, d e, and f).

(**g**) Immunoblot analysis. The hepatocytes described in (a) were cultured in regular medium. Cell lysates were prepared and subjected to immunoblot analysis with the indicated antibodies. Data shown are representative of three separate experiments. Bar graphs indicate the quantitative densitometric analysis of the indicated proteins relative to whole proteins estimated by Ponceau-S staining (n = 3). Data are means ± s.e. *$P < 0.05$, and ***$p < 0.001$ as determined by two-sided Welch's *t*-test. Source data are provided as a Source Data file.

(**h**) Long-lived protein degradation assay. Primary hepatocytes described in (a) were isolated and labeled with [^{14}C] leucine for 24 hr, and degradation of long-lived protein in deprived and non-deprived conditions was measured. E64d, pepstatin A and ammonium chloride (E64d/Pep+AC), or lactacystin was added as indicated. Bar graphs indicate the quantitative degradation rate of long-lived proteins (% of total per hour) (n = 3). Data are means ± s.e. *$p < 0.05$, **$p < 0.01$, and ***$p < 0.001$ as determined by two-sided Welch's *t*-test. Source data are provided as a Source Data file.

Supplementary Table 1

Primer sequences used for RT qPCR.

Gene	Left	Right
MmGus	GATGTGGTCTGTGGCCAAT	TGTGGGTGATCAGCGTCTT
MmNqo1	AGCGTTCGGTATTACGATCC	AGTACAATCAGGGCTCTTCTCG
MmGclc	GTGGACGAGTGCAGCAAG	GTCCAGGAAATACCCCTTCC
Mmp62	GCTGCCCTATACCCACATCT	CGCCTTCATCCGAGAAAC
MmGstm1	CTACCTTGCCCGAAAGCAC	ATGTCTGCACGGATCCTCTC
