# Supplementary material for: p62/SQSTM1-droplet serves as a platform for autophagosome formation and anti-oxidative stress response
Source: Nat Commun. 2021 Jan 4;12:16. doi: 10.1038/s41467-020-20185-1 (PMC7782522; doi:10.1038/s41467-020-20185-1)
Supplement: Supplementary file 14 — Description of Additional Supplementary Files [file 41467_2020_20185_MOESM14_ESM.docx]

Description of additional supplementary information

Title: Supplementary Movie S1

Description: Time-lapse video microscopic analysis of p62-structures labelled with GFP-p62 in Huh-1 cells.

Title: Supplementary Movie S2

Description: Time-lapse video microscopic analysis of purified p62-positive structures labelled with GFP-p62 after the treatment of 1,6- hexanediol.

Title: Supplementary Movie S3

Description: Time-lapse video microscopic analysis of p62-positive structures labelled with GFPp62 after photobleaching.

Title: Supplementary Movie S4 and S5

Description: Time-lapse video microscopic analysis of the dynamics of GFP-p62-positive structures in p62-GFPKI/+ (Movie S4) and Atg7-/-; p62-GFPKI/+ MEFs (Movie S5) after removal of As[III].

Title: Supplementary Movie S6

Description: Time-lapse video microscopic analysis of structures labelled with mCherry-p62 in HeLa cells expressing HyD-LIR-Venus by adenovirus system. HeLa cells were infected with adenovirus HyD-LIR-Venus for 30 hr and then transfected with mCherry-p62 for the labelling of p62-gels. 18 hr after the transfection, the cells were observed.

Title: Supplementary Movie S7

Description: Time-lapse video microscopic analysis of structures labelled with GFP-Keap1 in Huh-1 cells.

Title: Supplementary Movie S8

Description: Time lapse microscopic analysis. Huh-1 cells were transfected with GFP-Keap1. 24 hr after the transfection, the structures positive for GFP-Keap1 were photobleached, and then time of fluorescent recovery was measured.

Title: Supplementary Movie S9

Description: Time lapse microscopic analysis. Huh-1 cells were transfected with GFP-Keap1. 24 hr after the transfection, the cells were cultured in regular medium in the presence of 10 μM KMN003 and observed.

Title: Supplementary Data1

Description: Sequential Window Acquisition of All Theoretical Mass Spectra analysis with livers of HyD-LIRflox/flox and HyD-LIRflox/flox; Alb-Cre mice. T-test p-values were determined by two-sided Unpaired student’s t-tests.

Title: Supplementary Data2

Description: Prediction of Nrf2-targets on proteins that increased in livers of HyD-LIRflox/flox; AlbCre mice by 1.5-fold and more.
