# Supplementary material for: p62/SQSTM1-droplet serves as a platform for autophagosome formation and anti-oxidative stress response
Source: Nat Commun. 2021 Jan 4;12:16. doi: 10.1038/s41467-020-20185-1 (PMC7782522; doi:10.1038/s41467-020-20185-1)
Supplement: Supplementary file 15 — Reporting Summary [file 41467_2020_20185_MOESM15_ESM.pdf]

nature research

Corresponding author(s): Masaaki Komatsu

Last updated by author(s): Nov 10, 2020

Reporting Summary

Nature Research wishes to improve the reproducibility of the work that we publish. This form provides structure for consistency and transparency in reporting. For further information on Nature Research policies, see our Editorial Policies and the Editorial Policy Checklist.

Statistics

For all statistical analyses, confirm that the following items are present in the figure legend, table legend, main text, or Methods section.

n/a	Confirmed	
☐	☒	The exact sample size (*n*) for each experimental group/condition, given as a discrete number and unit of measurement
☐	☒	A statement on whether measurements were taken from distinct samples or whether the same sample was measured repeatedly
☐	☒	The statistical test(s) used AND whether they are one- or two-sided<br>*Only common tests should be described solely by name; describe more complex techniques in the Methods section.*
☒	☐	A description of all covariates tested
☒	☐	A description of any assumptions or corrections, such as tests of normality and adjustment for multiple comparisons
☐	☒	A full description of the statistical parameters including central tendency (e.g. means) or other basic estimates (e.g. regression coefficient) AND variation (e.g. standard deviation) or associated estimates of uncertainty (e.g. confidence intervals)
☐	☒	For null hypothesis testing, the test statistic (e.g. F, t, r) with confidence intervals, effect sizes, degrees of freedom and P value noted<br>*Give P values as exact values whenever suitable.*
☒	☐	For Bayesian analysis, information on the choice of priors and Markov chain Monte Carlo settings
☒	☐	For hierarchical and complex designs, identification of the appropriate level for tests and full reporting of outcomes
☒	☐	Estimates of effect sizes (e.g. Cohen's *d*, Pearson's *r*), indicating how they were calculated

Our web collection on statistics for biologists contains articles on many of the points above.

Software and code

Policy information about availability of computer code

Data collection	The data were collected using LightCycler® 480 Software version 1.5.1 (Roche Applied Science), Multi Gauge software V3.2 (FUJIFILM Corporation), FV10-ASW 04.01 (Olympus), FV31S-SW 2.4.1.198 (Olympus), SerialEM version 3.3.1 (https://bio3d.colorado.edu/SerialEM/), Integrative Genome Viewer version IGV 2.3.65 (http://software.broadinstitute.org/software/igv/), and BD FACSDiva software v8.0 (BD BioSciences).
Data analysis	The data were analyzed using TrakEM2 software version 1.0a 2012 -07-04, SerialEM version 3.3.1, IMOD software package version 4.7.3, Microscopy Image Browser version 2.651/25.04.2020, 3D Slicer software (https://www.slicer.org/), ImageJ (the core package in Fiji) version ImageJ 1.53c, Corel draw version 19.0.0.328, ProteinPilot 5.0.2. software (Sciex), SWATH variable window calculator tool version 1.0 (Sciex), PeakView 2.2 (Sciex), FV10-ASW 04.01 (Olympus), FV31S-SW 2.4.1.198 (Olympus), Multi Gauge V3.2 (FUJIFILM Corporation), DAVID v6.8 (Laboratory of Human Retrovirology and Immunoinformatics (LHRI)), Microsoft Excel version16.42 (Microsoft), and Adobe Photoshop CS6 or 2021 v25.0 (Adobe).

For manuscripts utilizing custom algorithms or software that are central to the research but not yet described in published literature, software must be made available to editors and reviewers. We strongly encourage code deposition in a community repository (e.g. GitHub). See the Nature Research guidelines for submitting code & software for further information.

Data

Policy information about availability of data

All manuscripts must include a data availability statement. This statement should provide the following information, where applicable:

- Accession codes, unique identifiers, or web links for publicly available datasets
- A list of figures that have associated raw data
- A description of any restrictions on data availability

Source data for Fig. 8c and Supplementary Dataset 1 were deposited. The accession numbers are PXD019492 (http://proteomecentral.proteomexchange.org/cgi/GetDataset?ID=PXD019492) for ProteomeXchange and JPST000830 (https://repository.jpostdb.org/entry/JPST000830) for jPOST. All figures and movies are available in figshare (URL). Source data are provided with this paper.

Field-specific reporting

Please select the one below that is the best fit for your research. If you are not sure, read the appropriate sections before making your selection.

☒ Life sciences ☐ Behavioural & social sciences ☐ Ecological, evolutionary & environmental sciences

For a reference copy of the document with all sections, see nature.com/documents/nr-reporting-summary-flat.pdf

Life sciences study design

All studies must disclose on these points even when the disclosure is negative.

Sample size	All experiments were repeated at least three times independently and s.e.m. were calculated from those data. The group sizes of the animals chosen are based on the numbers we used for previous publications, and the sizes are large enough to determine statistically significant effects.
Data exclusions	No data were excluded intentionally.
Replication	All experiments were repeated at least three times independently. Replicated experiments are explained in the text.
Randomization	No randomization of mice. Mice analyzed were littermates and sex-matched whenever possible. Samples were allocated based on genotypes or conditions. All cells from multiple dishes were combined and then plated into wells that were treated with various stresses or reagents. Thus, all treatment groups came from the same cell stock.
Blinding	Investigators were not blinded to mouse genotypes during experiments. Data reported for mouse experiments are not subjective. The investigators did not consider blinding necessary because all groups, including cellular experiments, were treated the same way.

Reporting for specific materials, systems and methods

We require information from authors about some types of materials, experimental systems and methods used in many studies. Here, indicate whether each material, system or method listed is relevant to your study. If you are not sure if a list item applies to your research, read the appropriate section before selecting a response.

Materials & experimental systems

n/a	Involved in the study	
☐	☒	Antibodies
☐	☒	Eukaryotic cell lines
☒	☐	Palaeontology and archaeology
☐	☒	Animals and other organisms
☒	☐	Human research participants
☒	☐	Clinical data
☒	☐	Dual use research of concern

Methods

n/a	Involved in the study	
☒	☐	ChIP-seq
☒	☐	Flow cytometry
☒	☐	MRI-based neuroimaging

Antibodies

Antibodies used	Antibodies against p62 (GP62-C, Progen Biotechnik GmbH, Heidelberg, Germany; 1:1000), S403-phosphorylated p62 (GTX128171, GeneTEex, CA, USA; 1:1000), NBR1 (4BR; Santa Cruz Biotechnology, Dallas, TX USA; 1:1000), LC3B (#2775, Cell Signaling Technology; 1:500), KEAP1 (10503-2-AP, proteintech, Rosemont, USA; 1:1000), Nqo1 (ab34173; Abcam, Cambridge, UK; 1:2000), Gclc (ab41463, Abcam; 1:500), GSTm1 (GSTM12-S, ALPHA DIAGNOSTIC INTERNATIONAL, San Antonio, USA ; 1:1000), and GFP (594, Medical and Biological Laboratories, Nagoya, Japan; 1:1000) were purchased from the indicated suppliers. Anti-S349-phosphorylated p62 polyclonal antibody (1:1000) was raised in rabbits by using the peptide Cys+KEVDP(pS)TGELQSL as an antigen. Blots were incubated with horseradish peroxidase-conjugated goat anti-mouse IgG (H+L) (Jackson ImmunoResearch Laboratories, Inc., 115-035-166;

1:10000), goat anti-rabbit IgG (H+L) (Jackson ImmunoResearch Laboratories, Inc., 111-035-144; 1:10000) or goat anti-guinea pig IgG (H+L) antibody (Jackson ImmunoResearch Laboratories, Inc., 106-035-003; 1:10000).
For immunofluorescence microscopy experiment, the samples were incubated with Goat anti-Guinea pig IgG (H+L) Cross-Adsorbed Secondary Antibody, Alexa Fluor 488 (A11073, Thermo Fisher Scientific), Goat anti-Mouse IgG (H+L) Highly Cross-Adsorbed Secondary Antibody, Alexa Fluor 647 (A21236, Thermo Fisher Scientific) and Goat anti-Rabbit IgG (H+L) Highly Cross-Adsorbed Secondary Antibody, Alexa Fluor 647 (A21245, Thermo Fisher Scientific) at a dilution ratio of 1:1000. For immunohistofluorescence microscopy experiment, the samples were incubated with Alexa647-conjugated donkey anti-guinea pig IgG (706-605-148, Jackson ImmunoResearch laboratories, Inc., West Grove, PA), and Alexa594-conjugated donkey anti-rabbit IgG (711-585-152, Jackson ImmunoResearch laboratories, Inc.) or Alexa594-conjugated donkey anti-mouse IgG (715-585-150, Jackson ImmunoResearch laboratories, Inc.).

Validation

Purchased antibodies were used as per manufacturer's recommendations. Anti-S349-phosphorylated p62 antibody was validated by previous our paper (Mol Cell. 2013 Sep 12;51(5):618-31.doi: 10.1016/j.molcel.2013.08.003. Epub 2013 Sep 5.)

Eukaryotic cell lines

Policy information about cell lines

Cell line source(s)

Huh-1 and HeLa cell lines were obtained from ATCC and JCRB Cell Bank, respectively. p62-deficient Huh-1, p62-deficient MEFs, p62-GFP KI MEFs and Atg7-/-; p62-GFPKI/+ MEFs were generated by us. Keap1-deficient MEF were gifted by Dr. Masayuki Yamamoto (Tohoku University) (Nat Genet. 2003 Nov;35(3):238-45.doi: 10.1038/ng1248. Epub 2003 Sep 28.).

Authentication

All cell lines were authenticated by STR profile.

Mycoplasma contamination

We verified that all cell lines used in this study were negative to mycoplasma by observation by fluorescence microscopy.

Commonly misidentified lines
(See ICLAC register)

None of the used cell lines is listed in ICLAC database.

Animals and other organisms

Policy information about studies involving animals; ARRIVE guidelines recommended for reporting animal research

Laboratory animals

Description of research mice used for experiments can be found in the relevant figure legends and Methods. 5-week-old male and female HyD-LIRflox/flox and HyD-LIRflox/flox; Alb-Cre mice were used. For the generation of Rosa26-loxP-stop-loxP-HyD-LIR-Venus mice (HyD-LIR-Venus mice), targeting vectors were electroporated into mouse RENKA ES cells, selected with G418, and then screened for homologous recombinants by Southern blot analysis. All mice housed in a specific pathogen-free room under temperature (23°C± 3°C),and humidity (40~60%) controlled conditions with 12/12h light/ dark cycle.

Wild animals

Wild-type mice were not used in this study.

Field-collected samples

Samples collected from the field were not used in this study.

Ethics oversight

Mice were housed in specific pathogen–free facilities, and the Ethics Review Committee for Animal Experimentation of Juntendo University approved the experimental protocol.

Note that full information on the approval of the study protocol must also be provided in the manuscript.
